# Neuroprotective potential of 17‐beta Estradiol: Targeting Inflammation and Mitochondrial Dysfunction in Alzheimer’s Disease

**DOI:** 10.1002/alz70859_099442

**Published:** 2025-12-25

**Authors:** Pranav Mishra, Ehsan Esfahani, Paul Fernyhough, Benedict C. Albensi

**Affiliations:** ^1^ Division of Neurodegenerative Disorders, St. Boniface Hospital Albrechtsen Research Centre, Winnipeg, MB Canada; ^2^ Department of Pharmacology & Therapeutics, College of Medicine, University of Manitoba, Winnipeg, MB Canada; ^3^ Department of Pharmaceutical Sciences, Barry & Judy Silverman College of Pharmacy, Nova Southeastern University, Fort Lauderdale, FL USA

## Abstract

**Background:**

Alzheimer's disease (AD) a neurodegenerative disorder affecting memory and cognition, is also linked to inflammation and mitochondrial dysfunction. Amyloid beta (Aβ), the hallmark of AD, exacerbates these processes by activating pro‐inflammatory NF‐κB, leading to chronic inflammation and metabolic defects. The risk of developing AD significantly increases with the loss of estradiol, during menopause and aging. E2, particularly its 17‐beta form, has demonstrated neuroprotective properties in various cell types by regulating mitochondrial proteins, reducing oxidative stress, and decreasing inflammation. Our work performed using primary cortical neurons have shown that E2 can enhance mitochondrial function, regulate NF‐κB DNA binding and activation, and also protect cells from Aβ‐mediated neurotoxicity. This goal of this study is to investigate the neuroprotective effects of 17‐beta estradiol (E2) against Aβ‐mediated neuroinflammation and mitochondrial dysfunction, potentially opening new avenues for therapeutic interventions towards AD.

**Method:**

Primary cortical neurons from C57BL/6 mouse embryos were cultured under defined conditions and treated with 10µM Aβ to induce AD‐like pathology. Additionally, 10nM E2 treatment was administered where mentioned. Protein expression was analyzed by western blotting. Cell viability, cell cytotoxicity, and inflammatory cytokines were assessed using MTT, LDH, and ELISA based assays using the manufacturers’ instructions. Mitochondrial function was evaluated with SeahorseXF24 bioanalyzer.

**Result:**

Aβ treatment decreased pAMPK (master regulator of metabolism) and PGC‐1α (master regulator of mitochondrial biogenesis) levels while activating pro‐inflammatory NF‐κB. Pretreatment with E2 effectively restored pAMPK and PGC‐1α levels, and prevented the Aβ‐induced decrease in mitochondrial function and ATP production. Additionally, E2 reduced pro‐inflammatory NF‐κB p65 DNA binding and inflammatory cytokine levels that had increased with Aβ treatment. LDH and MTT assays revealed that E2 attenuated Aβ‐induced neurotoxicity, highlighting its protective effect against AD like pathology.

**Conclusion:**

Our findings so far demonstrate that E2 exerts significant neuroprotective effects against Aβ in primary cortical neurons. E2 effectively mitigates Aβ‐mediated mitochondrial dysfunction, inflammation, and neurotoxicity by restoring key regulatory proteins, reducing inflammatory markers and, enhancing mitochondrial function. These findings suggest that E2 could potentially serve as a therapeutic agent against AD, addressing both mitochondrial dysfunction and neuroinflammation.

